# Exosome Enveloped by Nano Lipid Particle a New Model for Signal Transducer and Activator of Transcription 3 Silencer Ribonucleic Acid Delivery System to a Glioblastoma Mice Model

**DOI:** 10.3390/cancers17101648

**Published:** 2025-05-13

**Authors:** Amir Monfaredan, Sena Şen, Alaviyehsadat Hosseininasab, Didem Taştekin, Ghazaleh Fazli, Hamza Uğur Bozbey, Nasrin Yousefi, Merve Hocaoğlu, Mustafa Oral Öncül, Rıdvan Seçkin Özen

**Affiliations:** 1 Department of Molecular Medicine, School of Advanced Technologies in Medicine, Tehran University of Medical Sciences, Tehran 1416753955, Iran; monfaredanamir@gmail.com; 2 Department of Basic Oncology, Oncology Institute, Istanbul University, 34093 Istanbul, Türkiye; sena.sen@istanbul.edu.tr; 3 GeneDia Life Science Company, Tehran 1416753955, Iran; shrzd.hsni@gmail.com; 4 Department of Clinic Oncology, Oncology Institute, Istanbul University, 34093 Istanbul, Türkiye; didem.tastekin@istanbul.edu.tr (D.T.); ugurbozbey@yahoo.com (H.U.B.); 5 Department of Developmental Biology, Science and Research Branch, Islamic Azad University, Tehran 1477893780, Iran; ghazalehfazli@yahoo.com (G.F.); ynasrin1990@yahoo.com (N.Y.); 6 Istanbul Genetics Group, Genetic Disorders Evaluation Center, 34365 Istanbul, Türkiye; merveh92@gmail.com (M.H.); ridvanseckinozen@gmail.com (R.S.Ö.); 7 Department of Infectious Diseases and Clinical Microbiology, Internal Medicine, Istanbul Faculty of Medicine, Istanbul University, 34093 Istanbul, Türkiye

**Keywords:** blood–brain barrier, exosome-based therapy, glioblastoma, STAT3 inhibition, siRNA delivery

## Abstract

**Background/Objectives:** Glioblastoma is a highly aggressive brain tumor with limited treatment options and poor prognosis. Signal Transducer and Activator of Transcription 3 (STAT3) plays a crucial role in glioblastoma progression, making it a promising therapeutic target. However, effective delivery of STAT3-silencing agents across the blood–brain barrier remains a significant challenge. This study evaluates the efficacy of Lipid Nanoparticles-EXOSOME COMPLEX STAT3-silencer treatment in reducing glioblastoma tumor growth by facilitating efficient small interfering RNA delivery and inhibiting STAT3 expression. **Methods:** A novel exosome-based drug delivery system was developed using NLP-EXOSOME COMPLEX nanoparticles loaded with STAT3-silencer siRNA. The therapeutic efficacy was assessed in vitro using human glioblastoma cell lines and in vivo using a glioblastoma mouse model. Tumor progression, STAT3 expression levels, and survival rates were analyzed. **Results:** The results demonstrated that Lipid Nanoparticles-EXOSOME COMPLEX effectively transported STAT3-silencer siRNA into glioblastoma cells, leading to significant STAT3 downregulation. This resulted in reduced tumor proliferation, increased apoptosis, and extended survival in vivo. The combination of lipid nanoparticles and exosomes provided a stable and efficient delivery mechanism with improved uptake and therapeutic efficacy. **Conclusion:** Lipid Nanoparticles-EXOSOME COMPLEX STAT3-silencer treatment offers a promising approach for targeted glioblastoma therapy by overcoming the blood–brain barrier limitations and enhancing STAT3 inhibition. Further research is necessary to optimize long-term efficacy, assess potential immune responses, and explore combinatory therapeutic strategies for improved patient outcomes.

## Simple Summary

Glioblastoma is an aggressive brain tumor with limited treatment options due to the difficulty of transporting drugs across the blood–brain barrier. This study investigates a novel approach using exosomes in combination with lipid nanoparticles to deliver RNA molecules that silence the STAT3 gene, which plays a key role in tumor growth. Using this targeted delivery system, we were able to reduce tumor cell proliferation and improve survival in a mouse model of glioblastoma in laboratory tests. The results suggest that exosome-based therapies could be a promising strategy for the treatment of glioblastoma. This research could contribute to the development of more effective and less invasive treatments for patients with this disease.

## 1. Introduction

Glioblastoma (GBM), classified as Grade IV by the International Health Organization, is the most common type of malignant brain tumor in adults [1]. Due to the widespread infiltration of brain tumors and the inability to achieve complete surgical resection, most tumors rapidly recur, leading to a poorer prognosis [2]. Despite receiving standard treatment with temozolomide, the median survival time for patients is at best 12–15 months, with a 5-year survival rate of approximately 5% [3,4]. Therefore, the development of new and effective treatments is of critical importance. GBM is classified into different subtypes based on its genetic characteristics [5]. In studies investigating the effects of different therapeutic approaches required for each patient and their impact on tumor progression, GBM was categorized into four subtypes based on its molecular features: neuron, astrocyte, oligodendrocyte, and cultured astrocytic gliomas [6,7]. Although specific subtypes vary among different research groups, the perineural subtype is generally recognized as having the most favorable prognosis while also being associated with the most severe symptoms.

Drug development for GBM has faced significant challenges due to the presence of the blood–brain barrier (BBB). The BBB is a barrier composed of tightly packed endothelial cells that form a selective and continuous layer, strictly regulating the entry and exit of molecules into brain tissue [8]. This barrier, consisting of endothelial cells with tight junctions, restricts the free diffusion of many substances [9]. Additionally, it is surrounded by pericytes and astrocytes, which help regulate permeability and maintain structural integrity [10]. The physicochemical properties of the BBB further complicate the drug development process [11]. The two main physicochemical properties of the BBB are lipophilicity and size [12]. Small lipophilic molecules or lipophilic drugs can easily cross the BBB through diffusion via the lipid bilayer of endothelial cell membranes [13]. In contrast, hydrophilic substances, including polar molecules and large proteins, struggle to pass through the BBB due to their low lipophilicity [14]. In addition to lipophilicity and size, other physicochemical properties such as hydrogen bonding capacity, charge, and polarity also influence BBB permeability [15]. Both invasive and non-invasive methods have been developed to enhance BBB permeability [16]. Among non-invasive techniques, nanoparticle-mediated drug delivery—particularly lipid-based, polymeric, and inorganic nanoparticles—has attracted significant interest. However, the development of these synthetic carriers remains challenging due to issues such as biotoxicity and poor BBB penetration capability [17].

Exosomes, natural Nano carriers with diameters of 30–100 nm, are extracellular vesicles composed of lipid bilayers that are naturally released into body fluids by various mammalian cells to facilitate cell-to-cell communication, making them a promising solution [18]. They have low biotoxicity and have been the subject of more than 19,000 scientific articles indexed in Scopus and more than 200 clinical studies on their potential as diagnostic and therapeutic candidates. However, despite this potential, there are currently no FDA-approved exosome products. The extraordinary ability of exosomes to penetrate biological barriers makes them highly effective Nano vehicles for drug delivery [19]. Advances in microfabrication technology have significantly improved the isolation of exosomes in recent years. However, each isolation method has its own advantages and limitations, and the most appropriate technique should be selected based on the starting material and the intended use of the isolated exosomes [20]. The charge of exosomes plays a crucial role in complexation with RNAi. The exosomal membrane carries a negative charge, which facilitates electrostatic interactions with NLP, enhancing the stability and efficiency of RNAi delivery [21]. Understanding these physicochemical properties is essential for optimizing exosome-based therapeutic platform. The oncogenic transcription factor Signal Transducer and Activator of Transcription 3 (STAT3) is a critical molecule that is frequently overexpressed and hyperactivated in GBM [22]. It is strongly linked to the most aggressive and drug-resistant subtype, known as the stromal subtype, which is responsible for promoting cell growth, survival, angiogenesis, and immune response [23]. The phosphorylation of various kinases is responsible for the activation of STAT3 [24]. In glioma, Serin727 (S727) phosphorylation is dependent on Tyrosine705 (Y705) phosphorylation, which is required for the maximum activation of STAT3 [25]. Activated STAT3 is involved in the expression of numerous genes that are crucial for cancer progression, including those involved in migration and invasion (Matrix Metalloproteinase 2 (MMP2), MMP9), epithelial–mesenchymal transition (EMT) properties, and preventing escape and attack [26]. STAT3 also forms dimers and induces the expression of its target genes. In addition to glioma, STAT3 is hyperactivated in various other types of human cancers [27]. Previously, it has been demonstrated [28] that increasing the activity of the STAT pathway could effectively reduce the proliferation and migration of glioma cells in vitro and extend the survival of mouse tumors in vivo. One approach for targeting overexpressed or abnormally activated oncoproteins in cancer treatment is to decrease their expression using RNA interference (RNAi) [29]. This method involves using small INTERFERing^®^ transfection reagent RNA (siRNA), which has been found to have less impact than traditional drug treatments due to its sequence-specific nature [30]. Another significant advantage of RNAi is its ability to selectively disrupt the function of elusive or limited drug targets, such as STAT3 and other oncogenic transcription factors [31]. However, the successful treatment of developing gliomas requires the development of siRNA formulations for direct delivery.

According to this issue, we have developed siRNA complexes using nanoscale NLP-EXOSOME COMPLEX-Exosome complexes for in vivo delivery of therapeutic siRNA. These complexes protect the siRNA molecules, facilitate their cellular distribution and intracellular release, and have the potential for clinical application. This study aims to provide an updated evaluation of the latest drug delivery techniques designed to overcome the BBB and the blood–brain tumor barrier (BBTB). Specifically, it will explore the biogenesis, composition, isolation, and therapeutic cargo loading methods of exosomes as promising, natural, and biocompatible nano-tools. Additionally, the current applications of exosomes in GBM treatment will be highlighted, and potential future opportunities for developing novel drug delivery strategies to bypass the BBB will be discussed. Although exosomes themselves function as vesicular lipid systems, their encapsulation in NLPs has been developed to increase their stability, reduce rapid sheddingl and improve targeted delivery to glioblastoma cells. By combining the natural ability of exosomes to target cells with the controlled drug release properties of NLPs, we aimed to optimize therapeutic efficiency while maintaining the structural integrity of the exosomal cargo.

## 2. Materials and Methods

### 2.1. Cell Culture Experiments

The U87MG human glioblastoma cell line was acquired from the GeneDia Culture Collection. These cells were maintained in DMEM, supplemented with 20% FBS and 1% penicillin/streptomycin, and kept at 37 °C in a 5% CO_2_ humidified atmosphere. The cultures were maintained in a humidified incubator at 37 °C and 5% CO_2_ and were examined monthly for mycoplasma contamination using the PCR Mycoplasma Test Kit II (AppliChem, Darmstadt, Germany) according to the manufacturer’s instructions. To extract exosomes, U87MG cells were first seeded in 15 cm dishes and treated with 0.1 mg mL^−1^ PMA in Medium for 24 h. The media were then substituted with RMPI Medium 1640 supplemented with 10 μg mL^−1^ DSPE-PEG2000-Mal for 48 h. The remaining supernatant was collected after centrifugation at 2000× *g* for 15 min, followed by filtration through a 0.22 μm membrane. The filtrate was then concentrated using a 100 kDa ultrafiltration tube at 2200× *g* for 20 min to obtain the crude extract. The exosomes were subsequently extracted from the crude extract using a Plasma/Serum Exosome Purification and RNA Isolation Mini Kit (Norgen Biotek Corp, Thorold, ON, Canada) according to the manufacturer’s protocols. All chemicals were of ultrapure grade and obtained from MERCK (Merck KGaA, Darmstadt, Germany) under laboratory-grade conditions.

#### 2.1.1. Intact Exosome Purification and Exosomal RNA Isolation Followed by Complementary DNA Synthesis

Exosome isolation and RNA extraction were performed using Plasma/Serum Exosome Purification and RNA Isolation Mini Kit (Norgen Biotek Corp, Thorold, ON, Canada) according to the manufacturer’s protocols. The total non-coding RNAs and small RNAs, such as miRNAs, were converted to the cDNA extraction kit (ABM good Cat# G902, Richmond, BC, Canada). The miRNA sample was prepared by mixing 2 µL of 5X poly (A) polymerase reaction buffer, 1.5 µL ATP (10 mM), µ mL MnCl_2_ (25 mM), 0.5 µL Poly (A) Polymerase, Yeast (1 µg/µL), and 2.5 µL of H_2_O. Then, the mixture was incubated for 30 min at 37 °C, and 2 µL of miRNA Oligo (dT) adapter (10 mM) was added to the rest of the material. The mixture was incubated for 5 min at 65 °C followed by cooling on crushed ice. Finally, 1 µL of dNTPs (10 mM), 4 µL of 5X RT buffer, 1 µL RTase (200 U/µL), and 2 µL H_2_O were added to the above mixture. The cDNA synthesis was performed by incubating the samples for 15 min at 42 °C and 10 min at 70 °C.

#### 2.1.2. siRNAs Sequences

Synthetic siRNAs were acquired from MWG (Metabion, Planegg, Germany) had the following sequences: for the animal model, STAT3-silencer-1: GUUCAUCUGUGUGACACCA (sense); STAT3-silencer-2: CCUUCGAAGAAUCAAGCAG (sense). For the U87 cell line for the animal model, STAT3-silencer-1: ACACUGUAUCAGCAUAGCC (sense); STAT3-silencer-2: UCUACGAAGAAUCAAGCAG (sense).

#### 2.1.3. Stability of siRNA

After 24 h of incubation, 0.1 nmol of unbound siRNA were exposed to 25 μL of human plasma. The resulting mixtures were subjected to electrophoresis on a 20% TBE PAGE gel at 90 V for 180 min in TBE running buffer. Following electrophoresis, the gel was treated with 2.5 μL of EVAgreen gold in 25 mL of TBE buffer for 30 min and then detected using Sage 6000 (Merck, Darmstadt, Germany).

#### 2.1.4. Preparation of Exo-STAT3 siRNA

For the preparation of Exo-STAT3siRNA, 0.5 nmol siRNA was added to 100 μL of exosomes (0.5 mg mL^−1^) and then sonicated using an ultrasonic homogenizer (Sonica, Tehran, Iran) with the following settings: ultrasonic power 20 W, 10 cycles of 20 s, and 100 s off. The mixture was then incubated for 30 min at 4 °C. After incubating the mixture for 24 h, 0.5 mg of siRNA was added, and the solution was then washed twice with 150 μL of PBS using a 100 kDa ultrafiltration tube to remove any unconjugated siRNA and unloaded siRNA.

#### 2.1.5. Characterization of Exosomes

To observe the morphology of Exo-SiRNA and Exosome, transmission electron microscopy (TEM) was performed using a Hitachi microscope in Tehran, Iran. For this, 2 μL of engineered and nude exosomes were placed on a carbon-coated copper grid and allowed to dry for 10 min. Then, 20 μL of ammonium molybdate negative stain solution was added and left to dry for another 10 min. To ensure an appropriate concentration for measurement, the engineered exosomes were diluted with PBS. The primary antibody used was CD63, while the secondary antibody was anti-mouse IgG conjugated with horseradish peroxidase. The proteins were detected using Western blotting.

#### 2.1.6. Lipid Nanoparticles (NLP) Containing Exo-siRNA Using Microfluidic Technology by INSIGHT Nano Synthesis

To achieve this, the control of total flow rate (TFR) was manipulated between 2 and 20 mL/min while varying the ILR (Ionizable Lipid Reagent) concentrations (2–10 mg/mL). The flow rate ratios of aqueous: solvent was then adjusted from 1:1 to 10:1, using 10 mg/mL ILR in 40% ethanol, while keeping TFR at 5 mL/min. The concentrations in our test always refer to the siRNA (1 μg/mL indicates 1 μg of siRNA in 1 mL of culture). Ionizable lipid reagents (ILRs) facilitate the formation of lipid nanoparticles by enabling pH-dependent structural changes that enhance siRNA encapsulation. These lipids remain neutral at physiological pH but become positively charged in acidic environments, improving siRNA loading efficiency and intracellular delivery. The resulting NLP product was collected in a 15 mL falcon tube, with the initial volume of 0.5 mL and the final volume of 0.05 mL of NP solution being separately disposed of. Once the NLP-EXOSOME COMPLEX was synthesized, a solvent exchange method was used to remove the ethanol and replace it with water. The NLP-EXOSOME COMPLEXs were then diluted and centrifuged three times at 1600× *g* for 30-min runs using Ultra Centrifugal Filters with a nominal molecular weight limit of 10,000 NMWL. The size and distribution of the NLP-EXOSOME COMPLEXs were measured in both distilled water and PBS using the Malvern Zetasizer instrument (Worcestershire, UK).

The exosomes were not simply loaded into the nanoparticles but were actively complexed with NLP. The mechanism of complexation involves electrostatic interactions between the negatively charged exosomal membrane and the cationic lipids in NLP. Hydrophobic interactions also play a role in stabilizing the complex, preventing lipid phase separation, and maintaining structural integrity. This complexation process ensures optimal encapsulation and stability of siRNA, facilitating efficient intracellular delivery.

#### 2.1.7. Nanoparticle Characterization

The determination of particle sizes and zeta potentials was carried out using the Brookhaven ZetaPALS system (Brookhaven Instruments, Holtsville, NY, USA) through photon correlation spectroscopy (PCS) and phase analysis light scattering (PALS). Data analysis was conducted using the software provided by the manufacturer, with the viscosity and refractive index of pure water at 25 °C applied as reference parameters. Particle size measurements were performed over five independent runs, each lasting one minute, and the results were reported as the intensity-weighted mean diameter obtained from multiple experiments. Zeta potential measurements were conducted over 10 runs, with each run consisting of 10 cycles, and calculations were performed using the Smoluchowski model. Additionally, the hydrodynamic diameters of nanoparticles were assessed using nanoparticle tracking analysis (NTA) on an Insight LM 20 HS instrument (Malvern), which was equipped with a 640 nm sCMOS camera and a temperature-controlled sample chamber, following previously established protocols.

#### 2.1.8. Uptake Mechanism Studies

U87MG cells were pre-treated with 0.2 mM amiloride hydrochloride, 10 mM methyl-β-cyclodextrin (M-β-CD), 1 mM sucrose, and 0.02 mM cytochalasin D for 24 h before being incubated with labeled Exo-siRNA at a density of ~500 cells per well. Following a 4 h incubation, the cells were trypsinized, centrifuged at 800× *g* for 2 min, washed with PBS, and then resuspended in 300 μL of PBS for analysis using flow cytometry.

#### 2.1.9. Assays for Silencing STAT3 In Vitro

In vitro STAT3 silencing assays were performed using Quantitative Real-Time PCR (QRT-PCR) to study the activity of Exo-STAT3siRNA. U87MG cells were seeded in a 12-well plate (2 × 10^5^ cells per well), and after 24 h, they were treated with Exo-STAT3siRNA (0.05 nmol siRNA) or PBS for 24 h. The cells were then washed with PBS, and total RNA was extracted using a Total RNA Kit (Norgen Biotek Corp, Thorold, ON, Canada). Reverse transcription was performed using HyperScript 1st Strand cDNA, and qPCR was carried out using Universal SYBR qPCR Mix. The mRNA levels of STAT3 were normalized to the endogenous housekeeping gene, GADPH.

#### 2.1.10. Cytotoxicity Assays Conducted in a Laboratory Setting

U87MG cells were cultured in a 96-well plate at a density of 2 × 10^5^ cells per well and allowed to attach for 24 h. Then, the cells were treated with various formulations, including Exo-STAT3 siRNA (0.09 nmol siRNA), NLP- Exo-STAT3 siRNA (0.09 nmol siRNA), Exo (control), NLP (control), siRNA (0.09 nmol), and PBS. After 48 h, 10 μL of CCK-8 solution and 100 μL of DMEM were added to the wells and incubated for 2 h at 37 °C. The resulting relative cell viability was measured using a microplate reader at 450 nm.

#### 2.1.11. Apoptosis Assays

In order to evaluate the effectiveness of Exo-STAT3siRNA as a therapeutic treatment, a 6-well plate was prepared with U87MG cells (5 × 10^5^ cells per well) and incubated for 24 h. Subsequently, the cells were exposed to Exo-STAT3 siRNA (0.09 nmol siRNA), NLP- Exo-STAT3 siRNA (0.09 nmol siRNA), Exo (control), NLP (control), siRNA (0.09 nmol), and PBS for 48 h. To determine the effects of the treatment, Annexin V-FITC/PI was administered to the U87MG cells, and their progress was monitored using flow cytometry.

#### 2.1.12. Western Blotting

The procedure for preparing protein lysates from Tu2449 cells and mouse brain tumors was performed using SDS-PAGE and Western blotting. The membranes were incubated in 5% BSA/TBS-Tween20 (TBS-T) at room temperature for 1 h to blocked. Antibodies were then added and incubated at 4 °C overnight in 5% BSA/TBS-T. Secondary antibodies, either goat anti-mouse (dilution 5:20,000, PADTAN danesh, Tehran, Iran), were applied and incubated at room temperature for 1 h. For the analysis of protein expression in human cell cultures, cells were seeded and transfected in 24-well plates. After 72 h, the culture medium was removed, and the cells were rinsed with phosphate-buffered saline (PBS). Cell lysis was performed by adding radioimmunoprecipitation assay (RIPA) buffer (50 mM Tris, pH 7.4; 150 mM NaCl; 1% Triton X-100; 0.5% sodium deoxycholate; 0.1% sodium dodecyl sulfate (SDS); 2.5 mM sodium pyrophosphate; 1 mM ethylenediaminetetraacetic acid (EDTA); and a protease inhibitor cocktail (EDTA-free, Merck, Darmstadt, Germany)) to each well. The plates were then incubated on ice for 10 min. The resulting lysate was transferred to microcentrifuge tubes and subjected to centrifugation at 10,000 rpm for 10 min at 4 °C. The supernatant was collected, and total protein concentration was quantified using the TAKARA Protein Assay (TAKARA, Shiga, Japan). For electrophoretic separation, the protein lysates were combined with a loading buffer (0.25 mM Tris-HCl, pH 6.8; 20% glycerol; 10% beta-mercaptoethanol; 8% SDS; 0.08% bromophenol blue) to achieve a final concentration of 1×. A total of 20 µg of protein was loaded onto 20% polyacrylamide gels and separated via SDS-polyacrylamide gel electrophoresis (SDS-PAGE). The resolved proteins were subsequently transferred onto a 0.25 µm polyvinylidene difluoride (PVDF) membrane (Merck Millipore, Darmstadt, Germany). The membrane was blocked for 40 min using a blocking solution containing 5% (*w*/*v*) non-fat milk powder in Tris-buffered saline with Tween 20 (TBST; 10 mM Tris-HCl, pH 7.6; 150 mM NaCl; 0.1% Tween 20). Following blocking, the membrane was washed with TBST and incubated overnight at 4 °C with primary antibodies diluted in 3% (*w*/*v*) milk powder in TBST. The antibodies used included anti-human STAT3 and anti-Actin (both from Thermo Fisher Scientific, Waltham, MA, USA). After washing in TBST, the membrane was incubated for 1 h with horseradish peroxidase (HRP)-conjugated goat anti-rabbit IgG (Cell Signaling Technology, CST, Danvers, MA, USA), diluted in 3% (*w*/*v*) milk powder in TBST. The membrane was then subjected to additional washing before further analysis. The presence of bound antibodies was detected using enhanced chemiluminescence (ECL) kits SuperSignal^®^ West Femto (Thermo Fisher Scientific, Waltham, MA, USA).

### 2.2. Animal Experiments

Intracranial implantation of tumor cells was conducted in the right striatum of B6C3F1 mice, each with an average body weight of 30 g, following an established protocol. In this procedure, a total of 50,000 viable Tu2449 cells, suspended in 1 µL of solution, were injected at a controlled flow rate of 0.5 µL/min. The injection site was positioned 3 mm deep, 1.5 mm posterior, and 2 mm lateral to the bregma. After one week of tumor cell implantation, the 10 mice were randomly assigned into two experimental groups. One group was administered NLP-EXOSOME COMPLEX STAT3siCtrl treatment, while the other received NLP-EXOSOME COMPLEX STAT3-silencer treatment. The application of NLP-EXOSOME COMPLEXs was done using the same drill hole in the skull as for tumor cell implantation, at a frequency of every third day, starting one week after tumor cell implantation. The NLP-EXOSOME COMPLEXs, equivalent to 0.5 g siRNA in 5 L, were the maximum possible amount that could be used in this experimental setting due to limitations in the total NLP-EXOSOME COMPLEX volume. Fourteen days post-tumor implantation, five mice from each experimental group were euthanized. Of these, two mice were allocated for histological analysis, while another two were designated for molecular analysis. Tumor tissues were bisected, with one portion rapidly frozen on dry ice for subsequent protein extraction, and the other preserved in RNAlater (Qiagen, Hilden, Germany) to facilitate RNA isolation. All intracranial injections were conducted under anesthesia, and treatments were administered without blinding. Histological analysis revealed that in the NLP-EXOSOME COMPLEX STAT3siCtrl and NLP-EXOSOME COMPLEX STAT3-silencer groups, at the designated collection time point or upon the onset of neurological symptoms indicative of tumor burden, animals were euthanized using cervical dislocation following the administration of a lethal dose of anesthetic. The brains were promptly extracted, rinsed with phosphate-buffered saline (PBS), and fixed in 4% paraformaldehyde (PFA) (Chemcruz/Santa Cruz Biotechnology, Dallas, TX, USA) for a duration of 2 to 5 days. Prior to paraffin embedding, the fixed brains were coronally sectioned into slices measuring 2–3 mm in thickness and subsequently aligned in a horizontal orientation. The sections were embedded in Embedding Matrix (Thermo Fisher Scientific, Waltham, MA, USA) for further analysis.

#### 2.2.1. Laser-Capture Microdissection

Laser capture microdissection (LCM) was performed on 10 µm-thick serial sections using MMI membrane slides (Molecular Machines & Industries, MMI, Heidelberg, Germany), which had been UV-irradiated prior to use. The dissected sections were collected onto MMI Isolation caps equipped with diffuser caps (MMI). Before initiating the microdissection process, tissue sections were dried at 37 °C for one hour and subsequently rehydrated through two sequential cycles of ethanol treatment, including 100% xylene, followed by 99%, 96%, and 70% ethanol. To ensure tissue integrity and localization, the first and last sections of each series were stained with hematoxylin and eosin (H&E), dehydrated, and mounted using mounting medium (Leica, Wetzlar, Germany).

#### 2.2.2. MRI Acquisition

Magnetic resonance imaging (MRI) was carried out using a 7 Tesla small-animal MRI system (Bruker Biospec, Ettlingen, Germany) to track tumor development and evaluate therapeutic efficacy. T2-weighted images were obtained through a spin-echo sequence with the following settings: repetition time (TR) of 3000 ms, echo time (TE) of 60 ms, slice thickness of 1 mm, a field of view measuring 20 × 20 mm^2^, and a matrix resolution of 256 × 256. Scans were acquired in the transverse (axial) plane to allow clear visualization of both tumor mass and associated edema within the cerebral hemispheres. During scanning, mice were anesthetized using 2% isoflurane in oxygen and securely positioned in a stereotaxic holder to minimize motion artifacts. Baseline MRI imaging was performed on day 15 post tumor inoculation. Tumor volumes were measured using ImageJ-154 software by delineating hyperintense regions on each MRI slice and summing across all sections.

#### 2.2.3. Determination of Tumor Volume

Tumor volume was calculated on GFP positive areas in coronal brain sections using the following formula: volume = 0.5 × length × width × height.

#### 2.2.4. QRT-PCR

The amplification was carried out for 40 cycles with a pre-incubation at 95 °C for 15 s, followed by 10 s at 95 °C, 10 s at 55 °C, and 10 s at 72 °C for each cycle. A melting curve was obtained by rapidly cooling down from 95 °C to 65 °C, followed by a 15 s incubation at 65 °C and heating up to 95 °C. To normalize for equal mRNA/cDNA amounts, parallel runs were conducted with actin-specific primer sets for each sample. The ΔΔCt method was used to determine target levels. The following primer sets were used: human M87 cell line STAT3 forward primer: 5′ GGAGAAACAGGATGGCCCAA ′3, reverse primer: 5′ ATCCAAGGGGCCAGAAACTG ′3. The following primer sets were used: Mus musculus STAT3 forward primer: 5′ GTGTGACACCATTCATTGATGC ′3, reverse primer: 5′ CTCACATGGGGGAGGTAGCA ′3. Samples from laser-capture-micro dissected formalin-fixed paraffin embedded (FFPE) tissues were combined, and RNA was extracted using the Arcturus^®^ Paradise^®^ PLUS FFPE RNA Isolation Kit (Thermo Fisher, Waltham, MA, USA). The extracted RNA was then used to synthesize cDNA using SuperScript IV Vilo (life technologies/Thermo Fisher, Waltham, MA, USA) following the manufacturer’s instructions. The qPCR was performed on a StepOne Plus System (Applied Biosystems, Waltham, MA, USA) using 20 µL Taqman Probes (Applied Biosystems, Waltham, MA, USA) and 2 µL Fast-Start Universal Probe Master Mix (Roche, Basel, Switzerland) with 2 µL of cDNA, using the standard setting. The gene expression values were normalized using the mean Ct value of two housekeeping genes, β-Actin and GAPDH.

### 2.3. Statistics

The statistical analysis for this study was conducted using GraphPad Prism 7 (GraphPad Software, Boston, MA, USA). The Kaplan–Meier survival curves were evaluated using the log-rank test, while all other experiments were subjected to a two-tailed t-test with equal standard deviations and without correction for multiple comparisons. The measurement of cell cycle was analyzed using a two-way ANOVA of matched samples with Dunnett’s multiple comparison test against NLP-EXO-STAT3siCtrl-treated cells. Unless specified, the data are presented as boxplots, with the minimum and maximum values depicted on either end, and the median value represented by a horizontal line. The calculated mean is indicated by a ‘*’ symbol.

## 3. Results

### 3.1. The Use of NLP-EXOSOME COMPLEX Nanoparticles Results in the Production of Reliable and Efficient Formulations for Delivering siRNA

**Figure 1:**
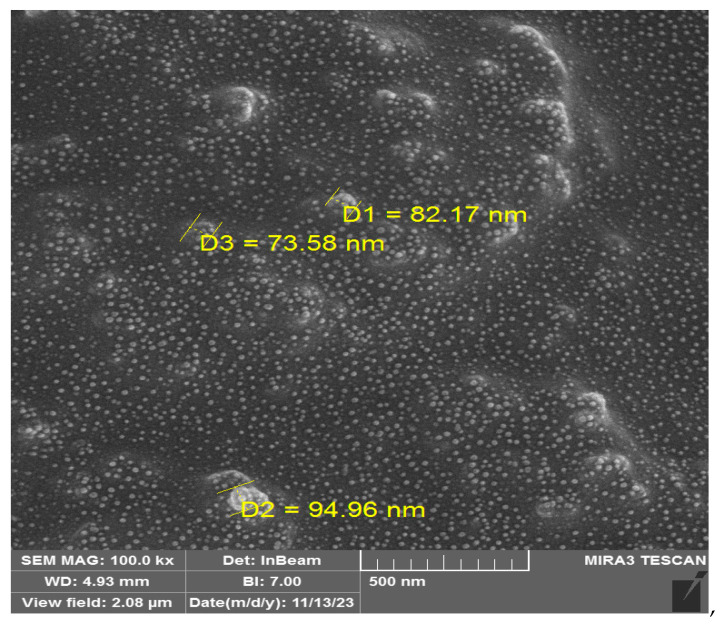
TEM images of Exo-SiRNA and nude exosomes. Representative TEM images show the characteristic morphology of both engineered (Exo-SiRNA) and unmodified (nude) exosomes. Samples were stained with ammonium molybdate and visualized using a Hitachi TEM microscope. Scale bar: 500 nm. (D: Diameter).

As previously mentioned, the combination of exosome containing siRNA (ExoSi) and ~5–10 kDa low-molecular-weight IONIZABLE CATIONIC LIPID (ICL) produced well-structured nanoscale complexes with sizes ranging from approximately 70–98 nm. The encapsulation of exosomes within NLPs was primarily mediated through electrostatic interactions between the negatively charged exosomal membrane and the cationic lipids of the NLPs. Hydrophobic interactions further stabilized the complex, preventing lipid phase separation and ensuring structural integrity. TEM and DLS analyses confirmed that the exosomes retained their morphological characteristics post encapsulation, indicating that NLP association did not compromise the lipid bilayer of the exosomes. The morphology of both Exo-SiRNA and nude exosomes was examined using TEM (Figure 1). TEM images confirmed the characteristic cup-shaped or spherical structure of the exosomes, consistent with previously reported exosomal morphology. Both engineered and unmodified exosomes exhibited a uniform size distribution, and no significant structural alterations were observed following siRNA loading. These findings indicate that the modification process did not compromise the exosomal integrity.

**Figure 2:**
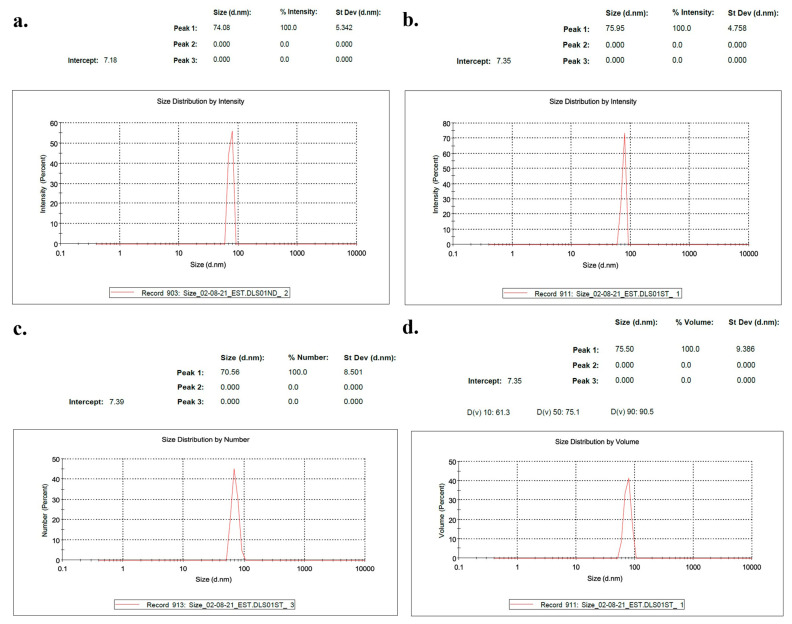
The NLP-EXOSOME COMPLEX nanoparticles’ reliable and efficient formulations for delivering siRNA. (**a**–**d**) The data presented demonstrate the characteristics and properties of nanoparticles.

The size of these complexes depended on the manufacturing process. INSIGHT measurements confirmed this, showing that the ExoSi complexes had a diameter of around 74 nm and a zeta potential of 24 mV (Figure 2a). When these complexes were mixed with ICL of similar size, slightly larger NLP-EXOSOME COMPLEXES (Figure 2b) were formed. The size order observed by INSIGHT was validated by dynamic light scattering (DLS), which also showed that the ExoSi complexes had a tendency to aggregate, resulting in smaller sizes (Figure 2c). However, the formation of NLP-EXOSOME COMPLEX inhibited this aggregation (Figure 2d). All DSL data are listed in the Supplementary File S1.

**Figure 3:**
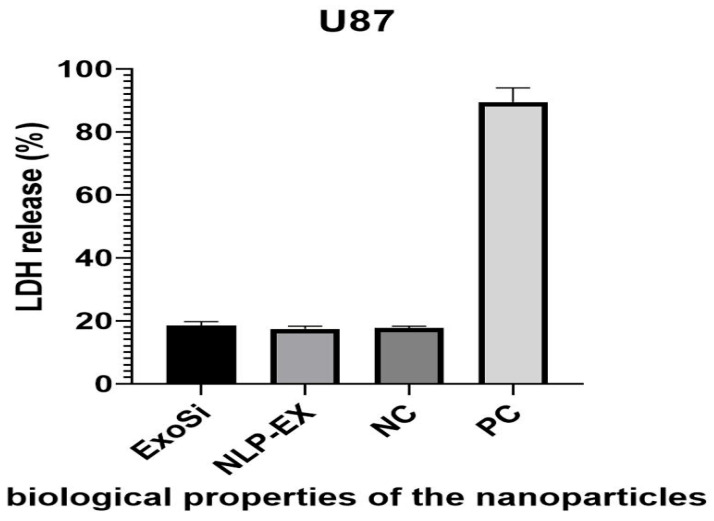
The biological properties of the nanoparticles were assessed, including the cytotoxicity, measured by LDH release assay. Each experiment was performed triplicate on biological samples, and the results of successful trials are presented. (PC: positive control, NC: negative control).

NLP-EXOSOME COMPLEX showed good biocompatibility in vitro, as demonstrated by the LDH release assay from U87 cells (Figure 3). The lack of cell damage during transfection suggests that NLP-EXOSOME COMPLEX is also safe for use in the central nervous system (CNS) in vivo, and the similar results seen with parental activity indicate that the degradation of NLP-EXOSOME COMPLEX and release of the complex does not cause toxicity.

### 3.2. The Inhibition of STAT3 Downregulate Tumorigenesis Both In Vitro and In Vivo

**Figure 4:**
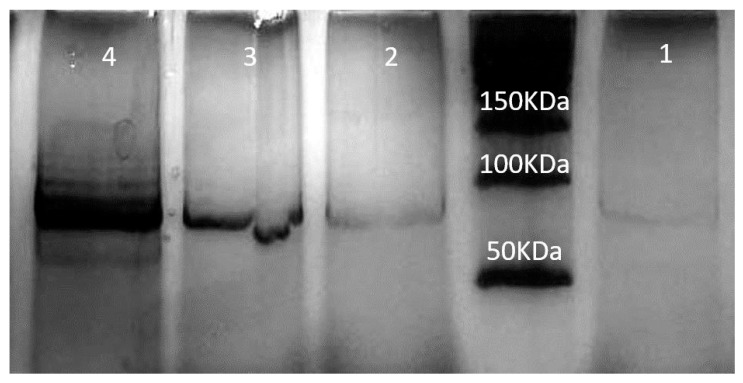
Western blot analysis of STAT3 expression in U87 cells transfected with control and STAT3-specific siRNAs. Western blot experiments were conducted on U87 cells after transfection with STAT3siCtrl, STAT3-silencer-1, or STAT3-silencer-2, as shown in rows 1–4, respectively.

**Figure 5:**
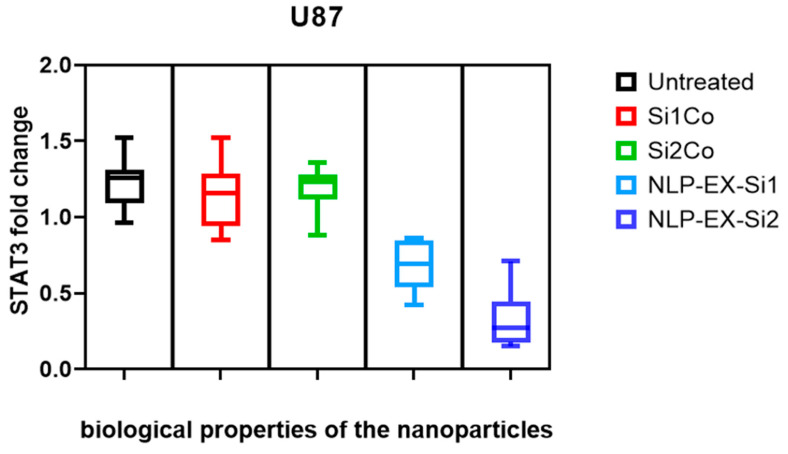
STAT3 expression analysis in U87 glioma cells post transfection with STAT3-specific siRNAs. Two STAT3-targeted qRT-PCR experiments were performed on the U87 human glioma cell line after transfection with either the control siRNA (STAT3-siCtrl) or two different siRNAs (STAT3-silencer-1 and STAT3-silencer-2). The expression of STAT3 was normalized using the ΔΔCt method and STAT3siCtrl-transfected cells were used as the control model.

In order to assess the potential therapeutic uses of NLP-EXOSOME COMPLEX-formulated siRNA both in vitro and in vivo, we specifically targeted STAT3, as it is known to be highly active in glioma and difficult to treat with traditional drugs. Benedikt Linder and et al.’s analysis of the TCGA dataset revealed that increased expression of STAT3 was correlated with survival rates among GBM patients, consistent with previous studies [32]. To validate these clinical findings in a laboratory setting, we utilized a human GBM cell line, U87 (Figure 4), in which we successfully downregulated STAT3 expression. This was achieved using two different siRNAs, with STAT3-silencer-2 proving to be more effective than STAT3-silencer-1. As a result, we were able to significantly reduce STAT3 mRNA levels in the cell line, and this was reflected in the reduced STAT3 protein expression (Figure 5).

**Figure 6:**
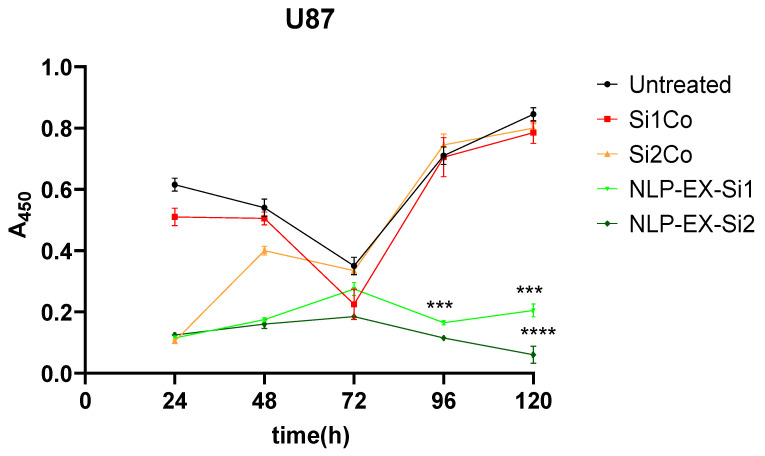
Proliferation assay of U87 glioma cells following STAT3 knockdown with siRNA and NLP-exosome delivery. Proliferation assays were performed on the U87 human glioma cell line using INTERFERin^®^ transfection reagent (untreated) and two different STAT3-silencers, 1 and 2 (Si1Co and Si2Co), and enveloped by NLP-EXOSOME(NLP-EX-Si1, NLP-EX-Si2). Statistical analysis showed significant differences (***: *p* < 0.001, (****: *p* < 0.0001) compared to Si1Co and Si2Co treatment.

Significantly, a distinct group of STAT3 signaling is typically observed in U87 cells, but this was not the case when targeting all three protein-coding sequences of STAT3 (NM_213662.1, NM_003150.3, and NM_139276.2) with two separate siRNAs. At 96 h after treatment, growth was reduced in U87 cell line, with STAT3-silencer-2 being more potent than STAT3-silencer-1. As a result, NLP-EXOSOME COMPLEX-mediated delivery of STAT3-silencer led to a strong inhibition of proliferation (Figure 6). All experiments in Figure 6 were conducted using three independent biological replicates, each performed in technical triplicates. Statistical significance between the Si1 and Si2 formulations was determined using an unpaired two-tailed *t*-test after confirming normality assumptions. Data are presented as mean ± SEM, and significance was set at *p* < 0.05.

The siRNA-mediated depletion of STAT3/Stat3 was effectively triggered, leading to the inhibition of tumor cell proliferation in vitro using the NLP-EXOSOME COMPLEX STAT3-silencer. Furthermore, U87 was validated as a viable model for investigating STAT3-targeted therapy.

It is important to note that the results presented in Figure 3 and Figure 6 assess different cellular responses under distinct experimental conditions. While Figure 3 evaluates cytotoxicity through LDH release, which directly measures cell death, Figure 6 focuses on cell proliferation, which reflects the ability of cells to divide and grow over time. The absence of significant cytotoxicity does not necessarily indicate that cell proliferation remains unaffected. Rather, our results suggest that while the tested formulations do not induce acute cytotoxic effects, they still exert an inhibitory effect on glioblastoma cell proliferation over time. These differences highlight the importance of using multiple assays to comprehensively evaluate the biological effects of the treatment.

To better understand the differences in therapeutic efficacy between SiRNA 1 and SiRNA 2, a sequence–specificity analysis was performed, and their STAT3 silencing efficiency was evaluated using qRT-PCR and Western blotting. The results indicated that while both siRNAs effectively reduced STAT3 expression, SiRNA 2 exhibited a more pronounced knockdown effect, leading to greater suppression of glioblastoma cell proliferation (Figure 5). This increased silencing efficiency translated into significantly improved survival rates in vivo, as observed in the Kaplan–Meier survival analysis (Figure 6). The superior performance of SiRNA 2 may be due to its enhanced stability, optimal incorporation into the RNA-induced silencing complex (RISC), or better targeting of STAT3 mRNA. These findings support the selection of SiRNA 2 as a more potent candidate for further development.

### 3.3. The Presence of NLP-EXOSOME COMPLEX Formulated Stat3 Silencer, Leads to an Increase in the Overall Survival Rate and a Decrease in Tumor Size in Mice with Glioma

**Figure 7:**
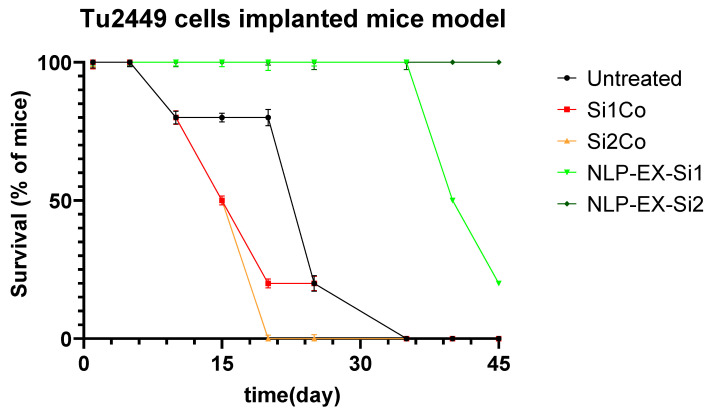
The Kaplan–Meier survival plot shows the survival of GBM mice implanted with Tu2449 and treated with NLP-EXOSOME COMPLEX STAT3siCtrl or NLP-EXOSOME COMPLEX STAT3-silencer. The in vivo experiment was carried out. Briefly, 20 mice were injected with 50,000 Tu2449 cells into the right striatum. After 7 days, the mice were divided into two groups (10 mice in each group) and were given intracranial treatment every 5 days. After 14 days, 4 mice were candidate for histological and molecular analysis, while the remaining mice were monitored for long-term survival. Mice without tumor development were excluded from the analysis. Between 19 and 50 days after NLP-EXOSOME COMPLEX STAT3-silencer treatment, the mice showed a significant difference in survival.

**Figure 8:**
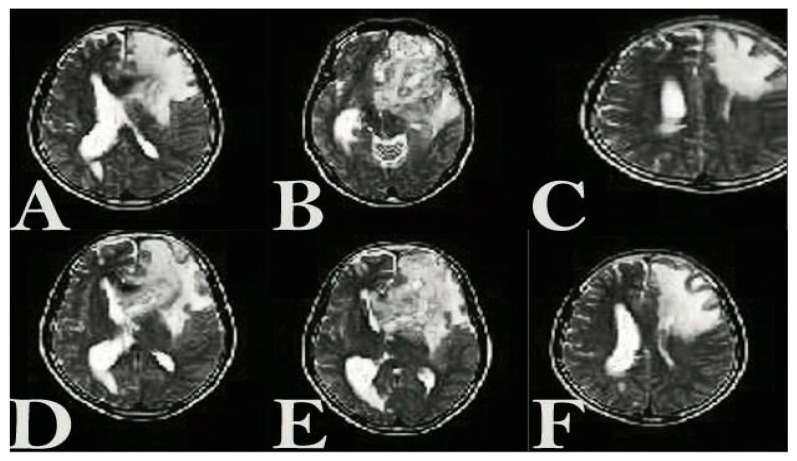
Transversal (axial) T2-weighted MRI images showing tumor localization and treatment response in glioblastoma-bearing mice. Hyperintense regions represent tumor masses, predominantly localized in the right striatum and cortex. (**A**) Model treated with the EXOSOME COMPLEX STAT3-silencer2 on day zero, (**B**) model treated with the EXOSOME COMPLEX STAT3-silencer2 on day 15, (**C**) model treated with the EXOSOME COMPLEX STAT3-silencer2 on day 30. (**D**) Model treated with the EXOSOME COMPLEX STAT3-control on day zero, (**E**) model treated with the EXOSOME COMPLEX-STAT3-control on day 15, (**F**) model treated with the EXOSOME COMPLEX-STAT3-control on day 30.

After allowing the tumors to grow for a week, 50,000 Tu2449 cells were implanted into the targeted layers of 20 mice. The mice were then separated into two groups, each consisting of 10 mice, and were given intracranial NLP-EXOSOME COMPLEX treatment every 5 days. After 2 weeks, four mice were candidate for histological and molecular analysis. Two tumors from the NLP-EXOSOME COMPLEX STAT3siCtrl and NLP-EXOSOME COMPLEX STAT3-silencer groups, which were programmed for histological analysis, did not grow and were therefore excluded from the study. Survival analysis (Figure 7) revealed that no mice died when treated with NLP-EXOSOME COMPLEX STAT3-silencer 2, while all 10 mice in the NLP-EXOSOME COMPLEX STAT3siCtrl group died. The median survival after NLP-EXOSOME COMPLEX STAT3-silencer treatment increased from 18 days to 50 days compared to NLP-EXOSOME COMPLEX STAT3siCtrl treatment. MRI analysis (Figure 8) confirmed that the mice who received NLP-EXOSOME COMPLEX STAT3-silencer had smaller tumors compared to those treated with NLP-EXOSOME COMPLEX STAT3siCtrl.

The rationale for incorporating exosomes into the ionizable lipid nanoparticle system was to enhance RNAi stability, biocompatibility, and targeted delivery to glioblastoma cells. While LNPs alone facilitate RNAi transfection, exosomes improve uptake efficiency and reduce immune clearance. Although this study primarily focused on intracranial administration as a proof-of-concept, future studies will investigate systemic delivery to validate BBB penetration.

### 3.4. The Presence of NLP-EXOSOME COMPLEX Resulted in a Decrease in the Expression of STAT3 Within the Tumor

**Figure 9:**
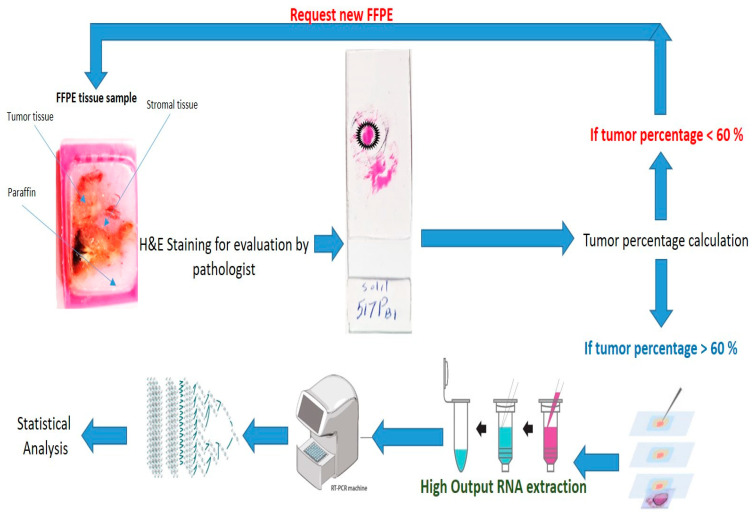
Schematic representation of the microdissection workflow from formalin-fixed paraffin-Embedded (FFPE) block selection to gene expression analysis via RT-PCR. The steps of implementing method microdissection are shown schematically from block selection to quantitative calculation of gene expression change of STAT3.

**Figure 10:**
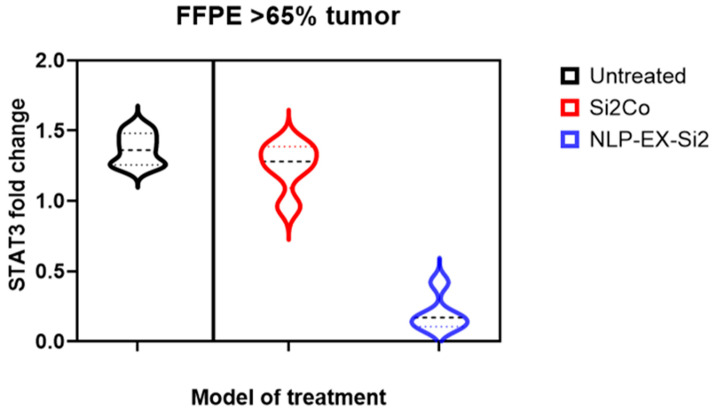
QRT-PCR analysis of tumor cells gene expressing tyrosine-phosphorylated STAT3 (mSTAT3) 15 days after implantation and treatment with NLP-EXOSOME COMPLEX STAT3siCtrl and NLP-EXOSOME COMPLEX STAT3-silencer2. Representative images are shown, the range is presented as a box, and the total sample is shown as a footnote; the horizontal line in the box indicates the mean, and the plus symbols indicate NLP-EXOSOME COMPLEX STAT3siCtrl or the mean of tumor cells at 15 days after tumor cell implantation after NLP-EXOSOME COMPLEX STAT3-silencer treatment.

After administering NLP-EXOSOME COMPLEX/STAT3-silencer treatment, we observed a significant increase in survival rate and decrease in tumor size. To further investigate this theory, we utilized laser capture microdissection to remove the central area of the tumor adjacent to the NLP-EXOSOME COMPLEX injection site (Figure 9). If any necrotic tissue was present, it was removed and discarded before isolating RNA from the remaining tumor tissue. QRT-PCR analysis was then performed on two samples from each group per working day, with yielding data for both Stat3 and the housekeeping gene GAPDH. Our results (Figure 10) showed a decrease in mRNA expression of Stat3 and its known target gene, GAPDH, after NLP-EXOSOME COMPLEX/STAT3-silencer treatment.

## 4. Discussion

Designing exosome packages with the ability to cross the BBB is critically important for several reasons, especially in the context of treating neurological diseases and delivering targeted therapies to the brain [33]. The BBB is a highly selective barrier that protects the brain from harmful substances but also significantly restricts the delivery of therapeutic agents [14]. Targeted delivery of therapeutics to the brain can reduce the required dosage and limit the distribution of drugs to other organs, thereby minimizing systemic side effects and toxicity [34]. Engineered exosomes can be designed to carry specific cargoes tailored to the molecular and genetic profiles of individual patients’ diseases, supporting the development of personalized medicine approaches. This precision targeting can improve therapeutic outcomes and reduce the likelihood of treatment resistance [35]. The encapsulation efficiency and stability of therapeutic cargo within exosomes are critical. Techniques to load exosomes with high concentrations of drugs, siRNAs, miRNAs, or proteins while maintaining their integrity and functionality are essential for effective therapy [36]. Despite extensive research efforts, GBM remains an incurable disease with a dismal prognosis for cancer patients [37]. The oncogenic transcription factor STAT3 plays a crucial role in regulating various drug resistance. High levels of STAT3 expression are linked to a more aggressive subtype of glioma, which is highly resistant to traditional treatments [38]. Furthermore, STAT3 has been found to control the stemness of glioma cells. While STAT3 is broadly implicated in glioblastoma, additional data on its role in perineural invasion have been included in the Section 4. While our model does not exclusively target perineural GBM, the findings are relevant due to STAT3’s general role in tumor progression. STAT3 activation contributes to multiple oncogenic pathways, including those involved in tumor invasion and resistance mechanisms, which may have implications for perineural glioblastoma as well.

Other investigations, as well as studies conducted by other groups, have demonstrated that both antibody targeting of STAT3 and stable lentiviral knockdown lead to significant anti-tumor effects in vitro and promote tumor growth in vivo. In order to promote the targeting of STAT3 in GBM, we opted to utilize a gene therapy strategy aimed at disrupting STAT3 expression. Our approach involved utilizing a unique method of utilizing siRNA packaged in NLP-EXOSOME COMPLEX/STAT3-silencer and evaluating its impact on cultured glioma cells, as well as in a syngeneic tumor transplantation model in mice, which closely mimics human gliomas. These tumors exhibited characteristics such as high mitotic activity, focal necrosis surrounded by pseudo palisade cells, and diffuse infiltration into the brain parenchyma. Through this technique, we were able to demonstrate that loading STAT3-siRNA onto NLP-EXOSOME COMPLEX resulted in the inhibition of glioma cell growth without causing any toxicity, as well as reduced tumor growth in vivo and improved outcomes for glioma-bearing mice. Additionally, multifunctional surfactants have been utilized as packaging agents to target Hif-1, and peptides have been used to dual target EGFR and Akt2. Several studies have explored exosome-based drug delivery for glioblastoma treatment, such as curcumin-loaded exosomes [39] and siRNA-loaded exosomes targeting EGFR [40]. Compared to these approaches, the NLP-EXOSOME COMPLEX STAT3-silencer system offers enhanced stability, improved BBB penetration, and targeted STAT3 inhibition, presenting a promising alternative for GBM therapy. This demonstrates that targeted delivery of siRNA to cancer cells can be achieved through various approaches, each with its own limitations. It is crucial for these delivery methods to effectively release siRNA in target cells and limit its degradation and capture by non-targeted cells or substances. A recent study also showed promising results with a combination drug containing an aptamer targeting PDGF beta and siRNA targeting STAT3, leading to reduced growth in both in vitro and in vivo glioma models, and utilized subcutaneous xenografts for in vivo experiments [41].

GBM is still one of the most aggressive and treatment-resistant brain tumors. The current standard treatment consists of surgical resection followed by radiotherapy and chemotherapy with TMZ. However, despite its widespread use, TMZ offers only a modest survival benefit, with median survival ranging from 12 to 15 months [42]. One of the major challenges associated with TMZ therapy is the development of resistance, primarily mediated by the DNA repair enzyme O6-methylguanine DNA methyltransferase (MGMT), which counteracts TMZ-induced DNA alkylation, thereby reducing its therapeutic efficacy [43,44]. In our study, we observed a significant reduction in tumor proliferation and an increase in apoptosis after treatment with the NLP-EXOSOME COMPLEX STAT3 silencer both in vitro and in vivo. Inhibition of STAT3 resulted in decreased viability of GBM cells and suppression of tumor growth, consistent with the known role of STAT3 as a critical oncogenic driver in GBM [22]. Comparing our results with those obtained with TMZ revealed several important differences. While TMZ exerted its cytotoxic effect primarily by inducing DNA damage, our siRNA-loaded exosome system specifically targeted STAT3, which plays a central role in GBM progression, immune evasion, and treatment resistance [45]. The in vitro data showed a significant reduction in STAT3 expression, resulting in impaired proliferation of U87 glioblastoma cells. These results are consistent with previous studies showing that inhibition of STAT3 reduces GBM cell survival and increases susceptibility to apoptosis [32]. Furthermore, in vivo results showed that the NLP-EXOSOME COMPLEX STAT3 silencer significantly prolonged the survival of tumor-bearing mice, with a remarkable reduction in tumor size observed by MRI analysis. By “long-term efficacy”, we refer to the ability of the NLP-EXOSOME COMPLEX to sustain STAT3 inhibition over an extended period and potentially prevent tumor recurrence. This is crucial for evaluating the therapeutic potential beyond initial treatment phases and assessing whether repeated dosing strategies or combination therapies could enhance durability. Further studies are needed to optimize formulation stability and evaluate immune responses over extended treatment periods. In contrast, although TMZ is effective in delaying tumor progression in vivo, its long-term efficacy is compromised by acquired resistance mechanisms, especially in tumors expressing high levels of MGMT [46]. The therapy aims to prolong survival rather than offer a complete cure. The transient effect of RNAi is a known limitation, but repeated dosing or combination with other therapeutic strategies may extend its benefits. Optimizing delivery mechanisms, enhancing nanoparticle stability, and exploring synergistic treatment combinations, such as immunotherapy or small molecule inhibitors, could further improve therapeutic outcomes. Future research should focus on addressing these challenges to maximize the translational potential of this approach.

Our study also encountered a similar limitation, as the small amount of siRNA perfusion may explain why there was only a mild decrease in STAT3 expression (and its target genes) in the tumors. Our findings indicate that the NLP-EXOSOME COMPLEX-STAT3-silencer delivery system only reached a small fraction of tumor cells, as evidenced by the reduction in STAT3-mRNA expression at the tumor site. However, due to the small sample size (five samples per group), a definitive conclusion cannot be drawn from these results. The study primarily focused on short- to mid-term outcomes, and the long-term efficacy and potential adverse effects require further clinical trial.

## 5. Conclusions

According to our results, most of the STAT3 activity values after NLP-EXOSOME COMPLEX STAT3-silencer treatment were found to be lower than the average value for NLP-EXOSOME COMPLEX STAT3siCtrl-transfected cells. Another significant finding is that nearly all of the cells treated with NLP-EXOSOME COMPLEX STAT3-silencer exhibited lower STAT3 activity values compared to the average value for NLP-EXOSOME COMPLEX STAT3siCtrl-transfected cells. This study highlights the efficacy of NLP-EXOSOME COMPLEX STAT3 silencer treatment in inhibiting STAT3 expression, reducing tumor proliferation and prolonging survival in GBM models. The results suggest that exosome-based siRNA delivery is a viable strategy for targeting GBM and represents a promising alternative to conventional therapies. However, further research is needed to optimize treatment efficacy, evaluate long-term safety, and explore potential combination approaches to improve therapeutic outcomes. With further advances in nanoparticle-based drug delivery systems, STAT3-targeted therapy could pave the way for more effective GBM treatment strategies in the future.

## Data Availability

The dataset used during the current study is available from the corresponding author upon reasonable request.

## Supplementary Materials

The following supporting information can be downloaded at: https://www.mdpi.com/article/10.3390/cancers17101648/s1, File S1: Size Characterization of NLP-EXOSOME COMPLEX Nanoparticles via DLS.
