# Supplementary material for: Exosome Enveloped by Nano Lipid Particle a New Model for Signal Transducer and Activator of Transcription 3 Silencer Ribonucleic Acid Delivery System to a Glioblastoma Mice Model
Source: Cancers (Basel). 2025 May 13;17(10):1648. doi: 10.3390/cancers17101648 (PMC12109797; doi:10.3390/cancers17101648)

Size Distribution Report by Intensity

v2.2

Malvern

Sample Details

**Sample Name:**	Size_02-08-21_EST.DLS01ND_ 2
**SOP Name:**	mansettings.nano
**General Notes:**	Sample was diluted 20 folds.

**File Name:**	Newfile-Askari-02-02-20.dts	**Dispersant Name:**	PBS
**Record Number:**	903	**Dispersant RI:**	1.332
**Material RI:**	1.38	**Viscosity (cP):**	0.9043
**Material Absorbtion:**	0.010	**Measurement Date and Time:**	Thursday, February 12, 2009 ...

System

**Temperature (°C):**	25.0	**Duration Used (s):**	10
**Count Rate (kcps):**	8.6	**Measurement Position (mm):**	5.50
**Cell Description:**	Clear disposable zeta cell	**Attenuator:**	11

Results

			Size (d.nm):	% Intensity:	St Dev (d.nm):
		**Peak 1:**	74.08	100.0	5.342
		**Peak 2:**	0.000	0.0	0.000
**Intercept:**	7.18	**Peak 3:**	0.000	0.0	0.000

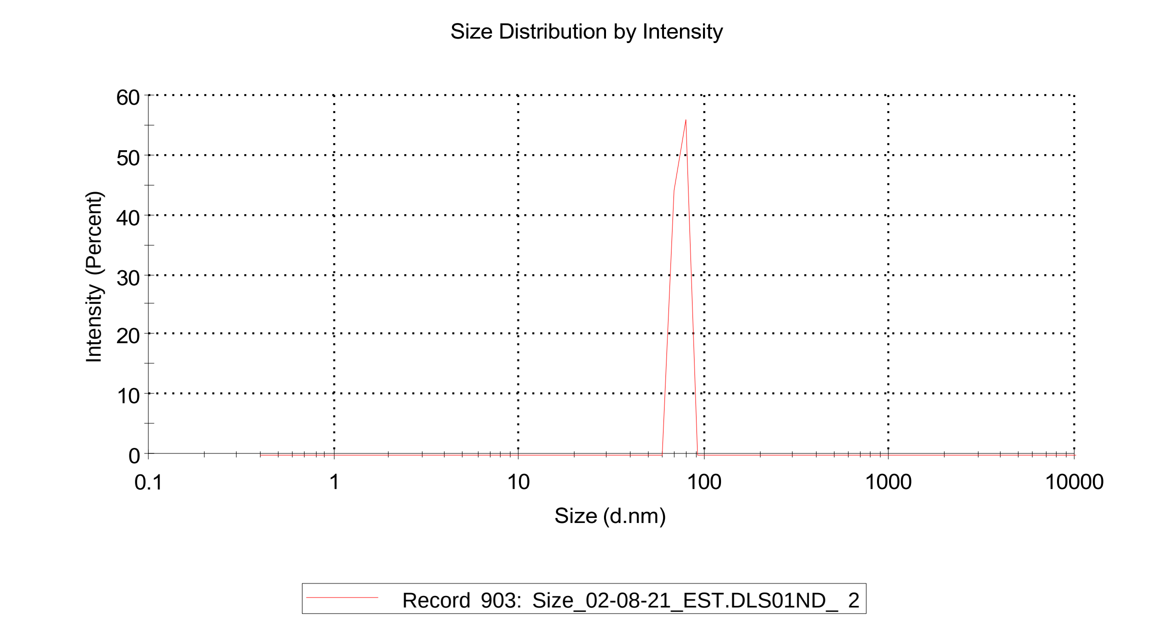

Size Distribution Report by Intensity

v2.2

Malvern

Sample Details

Sample Name: Size_02-08-21_EST.DLS01ST_ 1

SOP Name: mansettings.nano

General Notes: Sample was diluted 20 folds.

**File Name:**	Newfile-Askari-02-02-20.dts	**Dispersant Name:**	PBS
**Record Number:**	911	**Dispersant RI:**	1.332
**Material RI:**	1.38	**Viscosity (cP):**	0.9043
**Material Absorbtion:**	0.010	**Measurement Date and Time:**	Thursday, February 12, 2009 ...

System

**Temperature (°C):**	25.0	**Duration Used (s):**	10
**Count Rate (kcps):**	19.6	**Measurement Position (mm):**	5.50
**Cell Description:**	Clear disposable zeta cell	**Attenuator:**	11

Results

		Size (d.nm):	% Intensity:	St Dev (d.nm):
	**Peak 1:**	75.95	100.0	4.758
	**Peak 2:**	0.000	0.0	0.000
**Intercept:** 7.35	**Peak 3:**	0.000	0.0	0.000

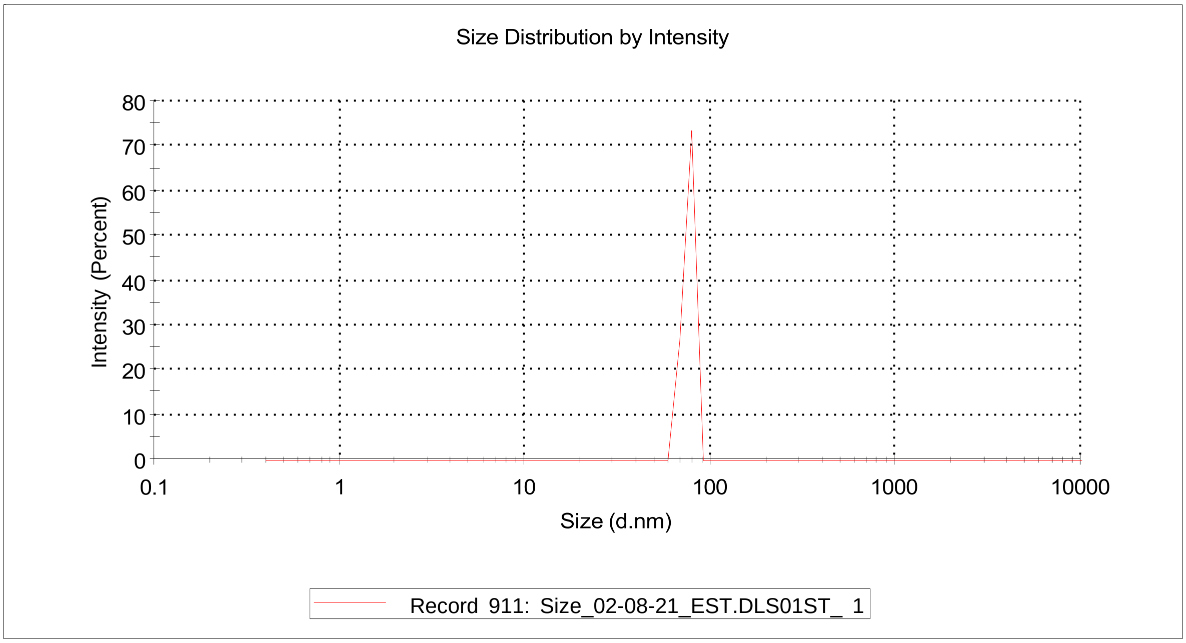

Size Distribution Report by Number

v2.2

Sample Details

Sample Name: Size_02-08-21_EST.DLS01ST_ 3

SOP Name: mansettings.nano

General Notes: Sample was diluted 20 folds.

File Name: Newfile-Askari-02-02-20.dts

Dispersant Name: PBS

Record Number: 913

Dispersant RI: 1.332

Material RI: 1.38

Viscosity (cP): 0.9043

Material Absorbtion: 0.010

Measurement Date and Time: Thursday, February 12, 200...

System

Temperature (°C): 25.0

Duration Used (s): 10

Count Rate (kcps): 15.4

Measurement Position (mm): 5.50

Cell Description: Clear disposable zeta cell

Attenuator: 11

Results

Intercept: 7.39

	Size (d.nm):	% Number:	St Dev (d.nm):
**Peak 1:**	70.56	100.0	8.501
**Peak 2:**	0.000	0.0	0.000
**Peak 3:**	0.000	0.0	0.000

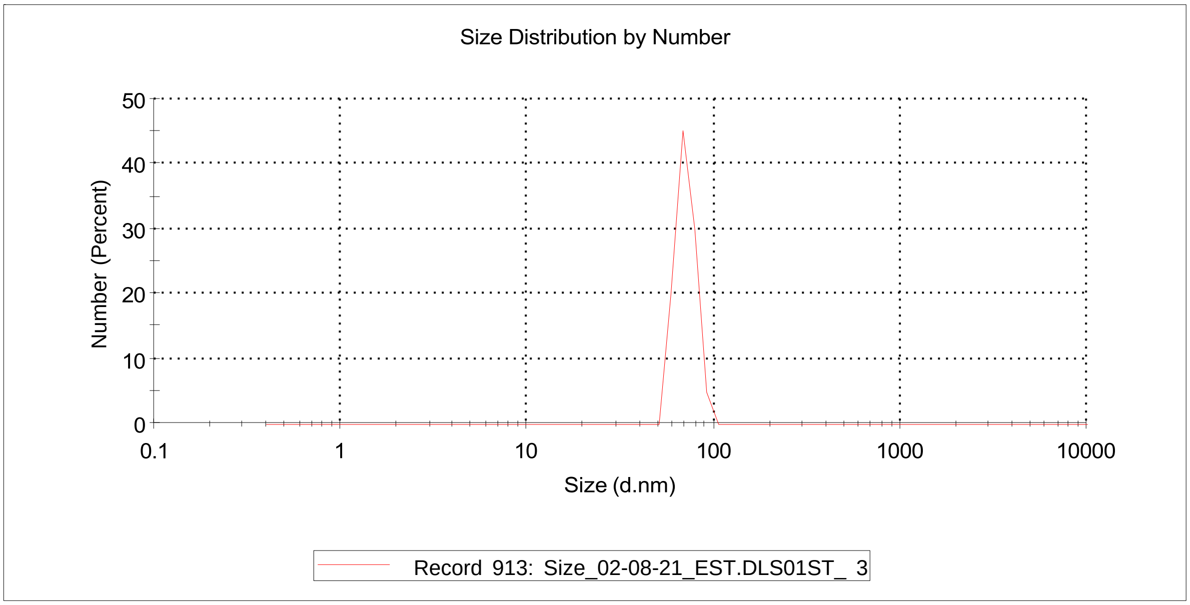

Size Distribution Report by Volume

v2.2

Malvern

Sample Details

Sample Name: Size_02-08-21_EST.DLS01ST_ 1

SOP Name: mansettings.nano

General Notes: Sample was diluted 20 folds.

File Name: Newfile-Askari-02-02-20.dts

Dispersant Name: PBS

Record Number: 911

Dispersant RI: 1.332

Material RI: 1.38

Viscosity (cP): 0.9043

Material Absorbtion: 0.010

Measurement Date and Time: Thursday, February 12, 2009 ...

System

Temperature (°C): 25.0

Duration Used (s): 10

Count Rate (kcps): 19.6

Measurement Position (mm): 5.50

Cell Description: Clear disposable zeta cell

Attenuator: 11

Results

	Size (d.nm):	% Volume:	St Dev (d.nm):
Peak 1:	75.50	100.0	9.386
Peak 2:	0.000	0.0	0.000
Peak 3:	0.000	0.0	0.000

Intercept: 7.35

D(v) 10: 61.3   D(v) 50: 75.1   D(v) 90: 90.5

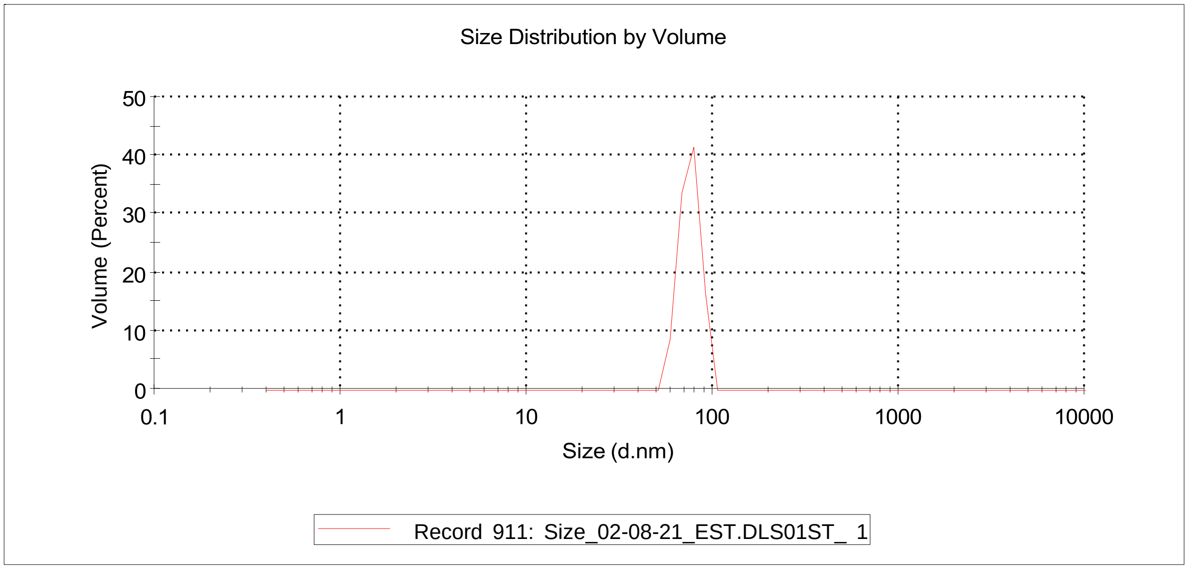

Supplement: Supplementary file 1 [file cancers-17-01648-s001.zip › cancers-3493422-supplementary.pdf]
